# Enhancement without a rationale? Black pepper extract (piperine) in dietary supplements and the future of formulation: a perspective from Japan

**DOI:** 10.3389/fnut.2026.1981406

**Published:** 2026-10-16

**Authors:** Masaki Kaneko

**Affiliations:** 1 KYB Medical Service Co., Ltd., Tokyo, Japan; 2 KYB Orthogenics Co., Ltd., Tokyo, Japan

**Keywords:** bioavailability enhancer, black pepper extract, cytochrome P450, dietary supplements, drug–nutrient interactions, formulation design, herb-induced liver injury, piperine

## Abstract

Bioavailability enhancers, most notably black pepper extract (piperine), are increasingly added to dietary supplements with claims of “improved absorption.” Piperine raises systemic exposure to co-administered compounds not by enhancing absorption but mainly by inhibiting drug-metabolizing systems, including CYP3A4, CYP2C9, and P-glycoprotein, and can therefore alter concentrations of concomitant medications, including drugs with a narrow therapeutic index. This Perspective argues that piperine is frequently included in multi-ingredient products without a defensible formulation rationale and, in the author's observation, now appears in Japan's practitioner-exclusive (clinic-dispensed) supplement market. A structured summary of 22 human pharmacokinetic studies shows that reports of enhanced nutrient absorption derive from manufacturer-affiliated studies of a few chemical forms; that trials without piperine-manufacturer involvement have not confirmed enhancement for curcumin or resveratrol at supplement doses; and that interaction studies with prescription drugs, mostly from groups with no commercial interest, repeatedly demonstrate altered drug exposure. This asymmetry is compounded by inadvertent supplement–supplement combinations and reports of turmeric–piperine-associated liver injury. The conditions under which piperine is a rational formulation choice are defined, and label warnings are endorsed as necessary but insufficient. “Practitioner-exclusive” labeling does not guarantee a sound formulation, and the good manufacturing practice requirements mandatory for Foods with Function Claims in Japan since September 2026 address manufacturing quality, not formulation appropriateness. Supplement formulation should be a deliberate, expert-driven discipline rather than an accumulation of trending ingredients; formulation rationale and interaction risk, not manufacturing quality alone, should become explicit considerations for clinicians, regulators, and industry.

## 1 Introduction

Dietary supplement use has expanded dramatically over recent decades, and product labels increasingly promise “enhanced absorption” or “improved bioavailability.” These claims are often based on the inclusion of black pepper extract, whose principal alkaloid, piperine, is marketed as a bioavailability enhancer (“bioenhancer”). The best-characterized application is the co-formulation of piperine and curcumin. In a widely cited study, 20 mg of piperine was reported to increase the systemic exposure to curcumin in humans by approximately 2,000% (1)—a figure that, as discussed in Section 5, was calculated against a baseline near the limit of detection and has not been confirmed at the piperine doses used in supplements in later trials conducted without involvement of the piperine-ingredient manufacturer.

At first glance, this appears to be an intelligent formulation strategy. This Perspective argues that the opposite is often true. Piperine is frequently added to multi-ingredient supplements without a valid rationale. Its mechanism of action carries an inherent risk of drug–nutrient interactions, and its widespread use exemplifies a broader deficiency in the formulation of dietary supplements. The aim is not to criticize any individual product or company but to use a widely encountered ingredient to illustrate a problem that, in the author's view, is common throughout the dietary supplement industry.

This concern is particularly relevant in Japan. Under the country's Foods with Function Claims framework, black pepper extract has increasingly appeared as an absorption enhancer, including, in the author's observation, in practitioner-exclusive (clinic-dispensed) products. Simultaneously, public attention to supplement safety intensified following health-damage cases linked to a functional food product in 2024, prompting regulatory reforms, including the introduction of mandatory good manufacturing practice (GMP) requirements, in force since 1 September 2026 (2). Therefore, Japan provides a timely setting to examine the gap between claims of enhanced absorption and a scientifically defensible formulation rationale.

## 2 Bioenhancer that acts by inhibiting metabolism

Piperine can increase the blood concentration of a co-administered compound by reducing the body's capacity to metabolize and eliminate that compound rather than facilitating intestinal absorption. For example, curcumin normally undergoes extensive hepatic and intestinal metabolism and conjugation, leaving little of the parent compound in systemic circulation (1). By inhibiting these elimination pathways, piperine can increase the amount of the compound that remains systemically available.

Mechanistically, piperine has been characterized as a relatively selective inhibitor of CYP3A4, whereas curcuminoids inhibit multiple cytochrome P450 enzymes, UDP-glucuronosyltransferase, and sulfotransferase enzymes (3). Inhibition of CYP2C9 has been inferred from interaction studies (4, 5), and piperine inhibits the efflux transporter P-glycoprotein (5). For curcumin, reduced glucuronidation and slower elimination have been proposed to explain the increase in plasma curcumin reported with piperine (1); whether this translates into a meaningful increase in unconjugated curcumin in humans is examined in Section 5.

In this context, the practical implication is clear: piperine does not selectively enhance the absorption of a single target nutrient. Although its enzyme inhibition is relatively selective, CYP3A4 and P-glycoprotein handle a large share of orally administered drugs, so inhibiting them relaxes shared metabolic and efflux “gatekeepers” for many compounds at once. Mechanistically, this is analogous to the food–drug interactions associated with grapefruit juice and St. John's wort, both of which modulate CYP3A4 activity (5, 6).

## 3 Clinically relevant drug interactions

If shared metabolic gatekeepers are relaxed, concomitant medications—not just the intended nutrients—may accumulate. Human studies support this concern. In healthy volunteers, 10 days of piperine (20 mg/day) increased the area under the concentration–time curve (AUC) of carbamazepine by approximately 48% (7). Similarly, in patients with epilepsy, a single dose of piperine significantly increased carbamazepine concentration and prolonged its half-life (8). Piperine also increased phenytoin exposure and peak plasma concentration in patients with epilepsy (9). For the nonsteroidal anti-inflammatory drug diclofenac, co-administration with piperine increased the AUC and half-life by approximately 66% and 34%, respectively, consistent with CYP2C9 inhibition (4). These are controlled pharmacokinetic studies in healthy volunteers and patients rather than case reports; the full set of human interaction studies is summarized in Table 1.

**Table 1:** Human pharmacokinetic studies of piperine co-administered with nutrients, phytochemicals, or prescription drugs, classified by funding or affiliation of the investigators.

Co-administered agent	References	Participants	Piperine regimen	Design	Effect on exposure	Funding/affiliation
A. Nutrients and phytochemicals						
Curcumin 2 g	Shoba et al. (1)	Healthy volunteers (n not reported in the accessible record)	20 mg, single dose	Single-dose comparison	Serum curcumin ↑ (reported +2,000%); curcumin alone near limit of detection	Piperine-ingredient manufacturer (author affiliation)
β-Carotene 15 mg/d	Badmaev et al. (18)	Healthy men with low baseline (*n* not reported in the accessible record)	5 mg/d, 14 d	Double-blind crossover	Serum β-carotene response ↑ (49.8 vs 30.9 μg/dL)	Piperine-ingredient manufacturer (author affiliation)
Coenzyme Q10 90–120 mg/d	Badmaev et al. (19)	12 healthy men	5 mg/d; single dose, 14 d, or 21 d	Double-blind, placebo-controlled	Single dose and 14 d: NS; 21 d (120 mg): ≈+30%	Piperine-ingredient manufacturer (funding declared)
Resveratrol 250 mg	Wightman et al. (23)	23 healthy adults (PK subset *n* = 6)	20 mg, single dose	Randomized double-blind placebo-controlled crossover	Plasma resveratrol and metabolites unchanged	No commercial affiliation identified (academic)
Curcumin 207 mg (8 formulations)	Flory et al. (20)	12 healthy adults	Piperine as adjuvant formulation	Randomized double-blind crossover	Piperine formulation NS vs native; micelles 57 ×, γ-cyclodextrin 30 ×	No commercial affiliation identified (academic)
Curcuminoids 1,515 mg with piperine (vs 4 formulations)	Fança-Berthon et al. (21)	30 healthy adults	Piperine–curcuminoid combination	Randomized open-label crossover	No significant difference vs standard extract	Competing-formulation manufacturer (author affiliation)
Curcumin 2,280 mg	Kroon et al. (22)	9 healthy men	20 mg, single dose	Crossover	No benefit; unconjugated curcumin < 2 nM in most participants	No commercial affiliation identified (academic hospital)
Turmeric extract 8 g (curcuminoids ≈ 3.6 g in the piperine-free arm, ≈ 6.8–7.5 g in the piperine arms)	Malhotra et al. (34)	14 healthy adults (5, 4 and 5 per arm)	Long-pepper extract (95% piperine) at 1% or 10% of the 8 g dose (≈ 76 and ≈ 760 mg piperine), single dose	Open-label randomized parallel-group, three arms	Curcuminoid AUC_0–24_ 1,480 (10% piperine) vs 25 (1% piperine) ng·h/mL; piperine-free comparator extract 1,458, from less than half the curcuminoid dose	Competing-formulation manufacturer (author affiliation)
B. Prescription drugs (interaction)						
Rifampicin	Zutshi et al. (24)	Patients with pulmonary tuberculosis (n not reported)	Co-administered	Comparative (details not available)	Rifampicin blood levels ↑	Patent-holding research group (public institute)
Phenytoin 300 mg	Bano et al. (25)	5 healthy volunteers	20 mg/d, 7 d	Crossover	AUC ↑; elimination t$\frac{1}{2}$ ↑	Patent-holding research group (public institute)
Propranolol 40 mg; theophylline 150 mg	Bano et al. (26)	6 healthy volunteers per drug	20 mg/d, 7 d	Crossover	C_max_ and AUC ↑ (both); t$\frac{1}{2}$ ↑ (theophylline)	Patent-holding research group (public institute)
Phenytoin 300 mg	Velpandian et al. (12)	6 healthy volunteers	Pepper-containing meal (culinary amount)	Crossover	Ka and AUC ↑; elimination delayed	No commercial affiliation identified (academic)
Phenytoin (steady state)	Pattanaik et al. (9)	20 patients with epilepsy	Single oral dose	Steady-state PK with vs without piperine	AUC and C_max_ ↑	No commercial affiliation identified (academic hospital)
Nevirapine 200 mg	Kasibhatta and Naidu (27)	8 healthy men	20 mg/d, 6 d + dosing day	Randomized placebo-controlled crossover	AUC ≈+170%; C_max_ ≈+120%	No commercial affiliation identified (academic hospital)
Carbamazepine (steady state)	Pattanaik et al. (8)	20 patients with epilepsy	20 mg, single dose	Steady-state PK with vs without piperine	AUC ↑; t$\frac{1}{2}$ ↑; C_max_ ↑ (500-mg group)	No commercial affiliation identified (academic hospital)
Midazolam, flurbiprofen, paracetamol	Volak et al. (31)	8 healthy volunteers	Curcuminoids 4 g + piperine 24 mg, 2 d	Randomized placebo-controlled crossover	No meaningful change	Piperine-ingredient manufacturer (co-author affiliation; academic lead)
Midazolam 10 mg	Rezaee et al. (28)	20 healthy volunteers	15 mg/d, 3 d	Placebo-controlled	Midazolam levels ↑; sedation prolonged	No commercial affiliation identified (academic)
Chlorzoxazone (CYP2E1 probe)	Bedada and Boga (29)	Healthy volunteers (n not reported in the accessible record)	20 mg/d, 10 d	Open-label sequential	C_max_, AUC, t$\frac{1}{2}$ ↑; clearance ↓	No commercial affiliation identified (academic)
Diclofenac 100 mg	Bedada et al. (4)	12 healthy volunteers	20 mg/d, 10 d	Open-label sequential	AUC +66%; C_max_ +64%; t$\frac{1}{2}$ +34%	No commercial affiliation identified (academic)
Carbamazepine	Bedada et al. (7)	12 healthy volunteers	20 mg/d, 10 d	Open-label sequential	AUC ≈+48%	No commercial affiliation identified (academic)
Fexofenadine 120 mg (P-gp probe)	Bedada and Boga (30)	12 healthy volunteers	20 mg/d, 10 d	Open-label sequential	AUC ≈+68%	No commercial affiliation identified (academic)
Tamoxifen 20–30 mg/d	Hussaarts et al. (32)	15 patients with breast cancer	Curcumin 1,200 mg + piperine 10 mg, 3 times daily, 28 d	Randomized three-period crossover	Endoxifen AUC −12.4% (−7.7% with curcumin alone)	No commercial affiliation identified (academic hospital; no conflicts declared)

AUC, area under the concentration–time curve; C_max_, maximum plasma concentration; Ka, absorption rate constant; *n*, number of participants; NS, not statistically significant; P-gp, P-glycoprotein; PK, pharmacokinetic; t$\frac{1}{2}$, half-life. Inclusion: human pharmacokinetic studies in which piperine or a piperine-containing preparation was compared with a control condition (placebo, no piperine, a piperine-free formulation, or a lower amount of piperine) and plasma exposure of a co-administered nutrient, phytochemical, or drug was reported. Attribution of an effect to piperine requires that the piperine-containing condition differ from its comparator only in the presence or amount of piperine. Three tabulated studies meet this condition only in part and are noted here: the piperine was given as a pepper-containing meal (12); the piperine arm also contained 4 g of curcuminoids (31); and the two piperine proportions differ also in curcuminoid dose, by about 9%, in the direction opposite to the reported effect (34). One further study is cited but not tabulated, because its piperine arm additionally contained lecithin in an unreported amount and was not compared statistically with the curcumin control (35). Only peer-reviewed full reports were eligible; conference abstracts and manufacturer summaries were excluded, as were animal studies, physiologically based pharmacokinetic models, and clinical-outcome trials without a piperine-free comparator. The table follows a PICO(T) structure: participants, intervention (piperine regimen), comparator and timing (design), and outcome (effect on exposure). Studies were identified by searching PubMed and Google Scholar (September 2026; terms: piperine AND [pharmacokinetics OR bioavailability] AND [human OR volunteers OR patients]) and by screening the reference lists of published reviews and of a systematic review (17); screening was performed by the sole author, and the summary is comprehensive to the author's knowledge but was not a formal systematic review. Funding/affiliation was classified from the authorship, affiliations, and funding or conflict-of-interest statements of each publication into four categories: piperine-ingredient manufacturer (authorship by, or funding from, a manufacturer of the piperine ingredient); competing-formulation manufacturer (authorship by a manufacturer of an alternative curcumin formulation); patent-holding research group (public research institute whose members hold a patent on piperine as a bioenhancer); and no commercial affiliation identified (academic, hospital, or public institutions without manufacturer authorship or declared manufacturer funding; “no conflicts declared” indicates an explicit statement in the publication). Supply of study material by a manufacturer was not assessed. Studies with both academic and manufacturer authorship carry both descriptors and are counted in the manufacturer category. A per-study appraisal of randomization, blinding, comparator structure, completeness of participant reporting, and sponsorship is provided as Supplementary Table S1. Within each panel, studies are ordered by year of publication. Effect sizes are as reported in the original publications, with percentage changes rounded and, where a publication gives means or ratios, derived from them; “n not reported” indicates that the number was not available in the accessible record.

Physiologically based pharmacokinetic modeling extends these observations. With piperine at 20 mg/day, the predicted AUC ratios exceeded 1.25 for several CYP3A4 substrates, including increases of approximately 34% for nifedipine, 35% for cyclosporine, and 59% for simvastatin (6). The greatest clinical concern involves drugs with a narrow therapeutic index, such as phenytoin and carbamazepine, for which even modest increases in systemic exposure can precipitate adverse effects, including nystagmus and ataxia (10). Similar caution is warranted for anticoagulants, digoxin, and immunosuppressants.

Two caveats should be noted. First, whether culinary amounts of black pepper produce clinically significant drug interactions remains unclear (5); the concern addressed here relates specifically to the pattern of supplement intake: isolated, highly concentrated piperine consumed as a daily bolus, often in combination with prescription medications (11). Even so, in one crossover study of six volunteers, phenytoin absorption and exposure increased when the drug was taken after a pepper-containing meal (12), although the clinical relevance of this finding is unknown. Second, several of these findings were derived from small, nonrandomized studies. Nevertheless, the consistency of the observed effects and their mechanistic plausibility support a cautious approach.

## 4 Inadvertent supplement–supplement combinations and hepatic exposure

A less-recognized risk arises from the concurrent use of multiple dietary supplements. Curcumin is a popular ingredient that is often included, sometimes in relatively small amounts, in multi-ingredient products marketed for joint, skin, gut, or liver support. Consequently, consumers who do not consider themselves to be “taking turmeric” may unknowingly ingest curcumin as part of a blended formulation. If they also take a separate supplement containing piperine, they are exposed, without knowing it, to the turmeric–piperine combination that has been associated with liver injury, at doses and durations for which, to the author's knowledge, no study has evaluated hepatic safety. Whether piperine meaningfully increases systemic exposure to curcumin in humans is uncertain (Section 5); the concern described here rests on clinical observations rather than on pharmacokinetic inference.

This concern is not merely theoretical. In a United States registry series of ten adjudicated cases of turmeric-associated liver injury, five patients were hospitalized and one died; three of the seven products analyzed also contained piperine, and the investigators raised the possibility that increasing combination with black pepper contributes to the rising number of cases, although causation is unproven and the absolute incidence appears to be low (13). Rising numbers of supplement-associated liver injury cases were reported at the 2026 Congress of the European Association for the Study of the Liver, where the turmeric–piperine combination was highlighted as a potential contributor (14), and individual cases of liver injury associated with piperine-containing turmeric supplements continue to be reported (15). For context, supplement-related liver failure leading to placement on the liver transplant waiting list in the United States has increased approximately eightfold over the past quarter century (16).

## 5 Enhancement without a target: what the human evidence shows—and does not show

This leads to the central issue: the rationale for including piperine in many products is unclear. Table 1 summarizes the 22 human pharmacokinetic studies of piperine identified by a structured, though not formally systematic, search (criteria and search terms in the legend to Table 1), grouped by the co-administered substance and classified by the funding or affiliation of the investigators, with a per-study appraisal of design and potential sources of bias in Supplementary Table S1; a quantitative synthesis of piperine–drug interaction trials in healthy volunteers has been published elsewhere (17).

Three patterns emerge. First, the studies reporting enhanced exposure of nutrients or phytochemicals were, with one 2025 exception discussed below, conducted or funded by the manufacturer of the piperine ingredient, enrolled small numbers of healthy volunteers where the number was reported, and used plasma concentration as a surrogate endpoint. In the most frequently cited study, curcumin given alone was largely undetectable, so the reported 2,000% increase was calculated against a baseline near the limit of detection of the assay (1). For β-carotene, a 14-day double-blind crossover study in healthy men with low baseline values reported a greater serum response with piperine (18). For coenzyme Q10, neither the single-dose nor the 14-day comparison reached significance, and only the 21-day, 120-mg arm showed an increase of approximately 30% (19). For vitamin B6, selenium, and vitamin C, the human data appear, to the author's knowledge, only as manufacturer summaries or conference abstracts rather than as peer-reviewed full reports. None of the studies identified here evaluated piperine in the multi-ingredient products in which it is most often marketed.

Second, human studies conducted without involvement of the piperine-ingredient manufacturer have generally not shown enhancement on the nutrient side. In three crossover trials of curcumin formulations, enrolling 12, 30, and 9 healthy adults (the last, men only), piperine-containing preparations did not differ significantly from native curcumin or standard extract (20–22)—although one of these trials was conducted by a manufacturer of a competing curcumin formulation (21)—whereas solubility-based formulations such as micelles and cyclodextrin complexes achieved substantially higher exposure, by 30-fold (γ-cyclodextrin) and 57-fold (micelles) in one of these trials (20); when unconjugated curcumin was measured separately, it remained below 2 nM in most participants even after 2,280 mg of curcumin with 20 mg of piperine (22). Earlier reports of enhancement, including the 2,000% figure, generally measured total (conjugated plus unconjugated) curcumin (22). For resveratrol, 20 mg of piperine did not alter the plasma concentrations of the parent compound or its metabolites in a pharmacokinetic subset of six participants (23). None of these trials was powered to exclude a modest effect, and the positive and negative curcumin studies also differ in era and analytical method, so improved measurement, rather than sponsorship alone, may explain part of the discrepancy.

Third, and in contrast, the interaction studies with prescription drugs, conducted largely by academic clinical pharmacology units, showed increased exposure to the co-administered drug in 12 of the 14 tabulated studies, most of them substrates of CYP3A4, CYP2C9, CYP2E1, or P-glycoprotein, including rifampicin, phenytoin, propranolol, theophylline, nevirapine, midazolam, chlorzoxazone, fexofenadine, diclofenac, and carbamazepine (4, 7–9, 12, 24–30), a pattern consistent with a meta-analysis of a small number of healthy-volunteer trials (17). The two exceptions are instructive. A 2-day course of a curcuminoid–piperine preparation did not alter midazolam, flurbiprofen, or paracetamol pharmacokinetics (31), whereas a 3-day course of piperine alone increased midazolam exposure (28); the reason for this discrepancy is unclear. And in patients with breast cancer, curcumin plus piperine reduced exposure to endoxifen, the active metabolite of tamoxifen, by 12.4% (7.7% with curcumin alone) (32); the direction of an interaction cannot be assumed to be an increase.

The asymmetry is the central point, and it should be stated with its qualifications. On the nutrient side, evidence of benefit is limited to manufacturer-affiliated studies of a few specific chemical forms; it has not been replicated independently for curcumin, the best-studied compound, at supplement doses, and has not been retested at all for β-carotene or coenzyme Q10. On the drug side, three of the 14 interaction studies come from the research group that holds a patent on piperine as a bioenhancer (24–26) and one has manufacturer-affiliated co-authors (31); the finding of increased exposure nevertheless rests on at least five further research groups with no identified commercial interest (4, 7–9, 12, 27–30), several of which used non-randomized, open-label designs. For drug substrates, therefore, this replication establishes piperine as an enhancer of systemic exposure; the unresolved question is whether the same holds for nutrients. Clinical trials of curcumin–piperine combinations have reported benefits (33), but because none of them compared the combination with curcumin alone, they cannot attribute any effect to piperine. Consequently, the presence of an absorption enhancer does not necessarily result in meaningful enhancement within a given product, whereas the potential for metabolic interactions remains. A 2025 three-arm study illustrates the point: curcuminoid exposure was much greater at a 10% than at a 1% proportion of long-pepper extract (≈760 vs. ≈76 mg piperine), yet the 10% arm did not differ from a piperine-free comparator extract given at less than half the curcuminoid dose (34). In practice, piperine is often included in formulations that contain no ingredient for which enhanced bioavailability has been convincingly demonstrated, yet the product is marketed with claims such as “formulated for improved utilization”—a practice that is difficult to reconcile with a coherent formulation rationale and more likely reflects the addition of a popular marketing ingredient.

## 6 When piperine is a rational formulation choice

None of the above implies that piperine should be excluded from dietary supplements. Its inclusion is scientifically defensible when several conditions are met together. First, the target ingredient should have a documented bioavailability limitation that is mechanistically addressable by inhibition of first-pass metabolism or efflux, as opposed to poor solubility, for which piperine is not the appropriate tool and solubility-based formulations perform better (20). Second, enhancement should have been demonstrated in humans for the specific chemical form and dose used in the product, preferably by investigators independent of the ingredient supplier and, where the compound is extensively conjugated, using the unconjugated fraction as the endpoint. Third, the intended population should be defined, and the product should not be positioned for routine daily use in individuals likely to be taking CYP3A4, CYP2C9, or P-glycoprotein substrates with a narrow therapeutic index. Fourth, the piperine dose should be the lowest shown to be effective, and the label should disclose its presence together with the relevant interaction precautions. A product that meets these conditions uses piperine as a pharmacological tool; a product that does not adds an interaction liability without a demonstrated benefit. On the evidence summarized in Table 1, few current nutrient combinations satisfy the second condition; the conditions describe the evidence a formulator should seek, not a standard that existing products already meet.

## 7 “Practitioner-exclusive” labeling and the limits of GMP

Labels such as “practitioner-exclusive” or “physician-dispensed” are marketing designations rather than regulatory categories. They may inspire confidence, but they do not, by themselves, indicate that a formulation is scientifically well-designed or that its safety has been established through premarket review. In Japan, as in many other jurisdictions, dietary supplements are not subject to the same premarket evaluation for safety and efficacy as pharmaceuticals. Instead, Foods with Function Claims are marketed under a notification system. Consequently, a restricted distribution channel does not guarantee the quality or appropriateness of a product formulation.

Products distributed through clinics are more likely to be used by patients who are also taking prescription medications. Incorporating an interaction-prone ingredient, such as piperine, into a baseline supplement intended for routine daily use in this channel therefore warrants careful justification. GMP-based manufacturing control became mandatory for Foods with Function Claims on 1 September 2026 (2). This is a welcome development, but GMP has limits. GMP ensures that a product is manufactured consistently, is free from contamination, and contains the ingredients listed on its label; it addresses the quality of manufacturing rather than the scientific rationale underlying the formulation and does not determine which ingredients should be combined, in what amounts, or for what purpose. Therefore, a product can be manufactured to a high standard while being formulated in a scientifically questionable manner. A cautionary label of the kind discussed in Section 8 would be a useful addition to such products; it would not, however, answer the question raised here, which is whether the formulation itself is appropriate for the population that this channel reaches.

## 8 Discussion

Piperine is not a problem when used with a clear understanding of its pharmacology; it is a legitimate formulation tool, and this Perspective does not argue for its prohibition. The problem lies in incorporating an ingredient—any ingredient—without a clear scientific rationale for its inclusion, at a specific dose, and in a particular combination. Formulating a dietary supplement is not simply a matter of assembling fashionable ingredients; it requires deliberate, evidence-based judgment grounded in nutritional science, pharmacology, and an understanding of potential *in vivo* interactions. In the author's view, supplement formulation should be recognized as an expertise-driven discipline with clearly defined professional responsibilities.

This Perspective has several practical implications. Clinicians should routinely inquire about dietary supplement use, including practitioner-exclusive products, when reviewing medications, given that piperine inhibits CYP3A4 and P-glycoprotein and, on the evidence of interaction studies, CYP2C9 (3–6) and may alter the systemic exposure of drugs with a narrow therapeutic index. Consumers should check product labels for “black pepper extract,” “Piper nigrum extract,” “piperine,” or branded piperine ingredients, and avoid unintentionally combining a piperine-containing supplement with a separate curcumin-containing product. Manufacturers—and the present author writes as one—should be prepared to justify the inclusion of every ingredient in a formulation.

Label warnings are part of the answer. A statement that individuals taking prescription medications, particularly anticonvulsants, anticoagulants, or immunosuppressants, should consult a clinician before using a piperine-containing product would be a low-cost, proportionate measure, and the author supports its adoption. Three considerations, however, indicate that labeling alone is insufficient. First, a warning presupposes that the direction and magnitude of an interaction are predictable, yet the tamoxifen study shows that co-administration of curcumin with piperine can decrease, rather than increase, exposure to an active metabolite (32). Second, a warning on one product cannot address exposure that arises from combining two products, neither of which is individually labeled as hazardous (Section 4). Third, in the practitioner-exclusive channel, the users most likely to be affected are those taking multiple medications, and a warning does not resolve the underlying question of why an interaction-prone ingredient was included in a product for which no benefit has been demonstrated. Labeling manages the risk; a formulation rationale determines whether the risk should exist at all.

Several research and policy directions follow. Independently funded human studies of piperine with commonly co-formulated ingredients, measuring the unconjugated fraction where relevant, are needed to establish which combinations, at which doses, produce meaningful enhancement; physiologically based pharmacokinetic modeling can prioritize the drug substrates of greatest concern (6). Regulators could require that any “enhanced absorption” claim be supported by human data for the specific form and dose in the product, and that the presence of piperine be declared on the label together with an interaction precaution. Pharmacovigilance systems should capture supplement–drug and supplement–supplement co-exposures, including those involving clinic-dispensed products. Ultimately, the ability to explain the rationale for every component distinguishes a thoughtfully formulated product from one that is merely well manufactured.

## Data Availability

The original contributions presented in the study are included in the article/Supplementary material, further inquiries can be directed to the corresponding author.

## Supplementary material

Supplementary Table S1 Per-study appraisal of design features and potential sources of bias in the 22 human pharmacokinetic studies summarized in Table 1.
