# Supplementary material for: Enhancement without a rationale? Black pepper extract (piperine) in dietary supplements and the future of formulation: a perspective from Japan
Source: Front Nutr. 2026 Oct 16;13:1981406. doi: 10.3389/fnut.2026.1981406 (PMC13719291; doi:10.3389/fnut.2026.1981406)
Supplement: Supplementary file 1 [file Table_1.docx]

**Supplementary Table S1**

**Per-study appraisal of design features and potential sources of bias in the 22 human pharmacokinetic studies summarized in Table 1**

Manuscript: "Enhancement Without a Rationale? Black Pepper Extract (Piperine) in Dietary Supplements and the Future of Formulation: A Perspective from Japan" (Manuscript ID 1981406). Author: Masaki Kaneko.

This appraisal was prepared in response to Reviewer 1’s request for a bias analysis. It is a structured, transparent appraisal rather than the application of a validated risk-of-bias instrument: the studies are pharmacokinetic rather than outcome trials, so tools designed for randomized clinical trials (RoB 2) or non-randomized studies of interventions (ROBINS-I) map onto them only in part. Each cell records what the publication states. "Not reported" means the item was not described in the record accessible to the author and is not evidence that the procedure was not performed. Because no quantitative synthesis is performed in the manuscript, the summary judgment indicates the confidence with which a single study’s point estimate can be read, not a score entering any pooled analysis. Judgments were assigned from the design, reporting and affiliation items, not from the direction of a study’s result; in panel A the two happen to correlate, because the studies reporting enhancement are also the older and less completely reported ones.

| **Study (ref.)** | **Randomization** | **Blinding** | **Comparator structure** | **Reporting of participants** | **Sponsorship / affiliation** | **Summary judgment and direction of concern** |
| --- | --- | --- | --- | --- | --- | --- |
| **A. Nutrients and phytochemicals** | | | | | | |
| Shoba 1998 (1) | Not reported | Not reported | Single-dose comparison with and without piperine; whether the comparison was within- or between-subject is not described | n not reported in the accessible record | Piperine-ingredient manufacturer (author affiliation) | Substantial concern. Sponsorship, absent randomization or blinding, and an effect size computed against a near-undetectable baseline all act in the direction of overstating enhancement. |
| Badmaev 1999 (18) | Not reported (crossover) | Double-blind | Double-blind crossover (β-carotene with piperine versus β-carotene alone) | n not reported in the accessible record | Piperine-ingredient manufacturer (author affiliation) | Moderate to substantial concern. Blinding reduces measurement bias; sponsorship and unreported sequence allocation act in the direction of overstating enhancement. Baseline serum β-carotene was low by design, which favors detection of a response. |
| Badmaev 2000 (19) | Not reported | Double-blind | Placebo-controlled, three durations (single dose, 14 d, 21 d) | n = 12 healthy men | Piperine-ingredient manufacturer (funding declared) | Moderate concern. The most completely reported of the three manufacturer studies, and the two shorter comparisons were null; the positive result rests on one arm, so selective emphasis among the reported arms, rather than measurement, is the relevant risk. |
| Wightman 2014 (23) | Randomized | Double-blind | Placebo-controlled crossover | n = 23; pharmacokinetic subset n = 6 | No commercial affiliation identified (academic) | Low concern for internal validity; imprecision is the limiting factor. A subset of six cannot exclude a modest effect, so the null result should not be read as evidence of absence. |
| Flory 2021 (20) | Randomized | Double-blind | Crossover, eight formulations including native curcumin | n = 12 healthy adults | No commercial affiliation identified (academic) | Low concern; no directional concern identified. The same study detected large increases for micellar and cyclodextrin formulations, which demonstrates assay sensitivity and strengthens the null result for the piperine arm. |
| Fança-Berthon 2021 (21) | Randomized | Open-label | Crossover against four other formulations, including a standard extract | n = 30 healthy adults | Competing-formulation manufacturer (author affiliation) | Moderate concern, and one of two studies in this table whose sponsor has an interest in the comparator rather than in piperine (the other is 34). The affiliation acts in the direction of understating piperine’s effect and is noted in the manuscript. |
| Kroon 2025 (22) | Not reported | Not reported | Crossover against other curcumin formulations | n = 9 healthy men | No commercial affiliation identified (academic hospital) | Moderate concern for design, low for conflict. The endpoint (unconjugated curcumin) is the most informative in the table; small n limits precision, and unreported allocation limits the appraisal of internal validity; direction indeterminate. |
| Malhotra 2025 (34) | Randomized | Open-label | Parallel group, three arms; the piperine proportion differs between two arms sharing the same base extract, and a third arm is a different piperine-free extract | n = 14 (5, 4 and 5 per arm) | Competing-formulation manufacturer (author affiliation) | Substantial concern. Parallel arms of four and five participants and an open-label design limit what the study can settle, and whether free or total curcuminoids were measured is not stated. The low-piperine arm showed almost no absorption from a larger curcuminoid dose than the high-piperine arm, which is hard to reconcile with the other entries here and which an artifact depressing that arm would also produce. The authors’ employer markets the piperine-free comparator, so the study’s headline result — parity between that product and the high-piperine formulation at less than half the curcuminoid dose — aligns with its commercial interest, and the direction of concern is toward understating piperine. The amounts compared are in any case far above supplement doses. |
| **B. Prescription drugs (interaction)** | | | | | | |
| Zutshi 1985 (24) | Not reported | Not reported | Comparative; details not available in the accessible record | n not reported | Patent-holding research group (public institute) | Substantial concern. The patent interest acts in the direction of overstating the interaction; the design cannot be appraised from the accessible record, so the direction of any design-related bias cannot be determined. |
| Bano 1987 (25) | Not reported | Not reported | Crossover, 7-day pretreatment | n = 5 healthy volunteers | Patent-holding research group (public institute) | Substantial concern. Very small sample, no reported randomization or blinding, patent interest. The patent interest acts in the direction of overstating the interaction; the direction of any design-related bias is indeterminate. |
| Bano 1991 (26) | Not reported | Not reported | Crossover, 7-day pretreatment | n = 6 per drug | Patent-holding research group (public institute) | Substantial concern, as above, with the same direction. Two drugs were studied in separate small groups. |
| Velpandian 2001 (12) | Not reported | Not reported | Crossover; piperine given as a pepper-containing meal | n = 6 healthy volunteers | No commercial affiliation identified (academic) | Moderate to substantial concern from size and unreported allocation, of indeterminate direction; no commercial interest identified. Cited in the manuscript for the culinary-exposure question, where its unusual exposure route is the point. |
| Pattanaik 2006 (9) | Not reported | Not reported | Within-patient comparison at steady state, with and without piperine | n = 20 patients with epilepsy | No commercial affiliation identified (academic hospital) | Moderate concern. If the order of the two conditions was not randomized, time-related change cannot be separated from the intervention, although steady-state dosing limits this; direction indeterminate. Conducted in the population of clinical interest. |
| Kasibhatta 2007 (27) | Randomized | Not reported (placebo-controlled) | Placebo-controlled crossover | n = 8 healthy men | No commercial affiliation identified (academic hospital) | Low to moderate concern; no directional concern identified. One of the three randomized crossover designs on the drug side (with 31 and 32); small n limits precision, but the reported effect is large. |
| Pattanaik 2009 (8) | Not reported | Not reported | Within-patient comparison at steady state, with and without piperine | n = 20 patients with epilepsy | No commercial affiliation identified (academic hospital) | Moderate concern of indeterminate direction, as for the 2006 study by the same group; the two share a design and cannot be treated as fully independent replications of each other. |
| Volak 2013 (31) | Randomized | Not reported (placebo-controlled) | Placebo-controlled crossover, 2-day course | n = 8 healthy volunteers | Piperine-ingredient manufacturer (co-author affiliation; academic lead) | Low concern for design. The affiliation would act in the direction of overstating an effect, and the result was null, so the affiliation does not explain the finding. This study differs from the 3-day piperine-alone study (28) in both preparation and duration, and the manuscript leaves the reason for the discrepancy open. |
| Rezaee 2014 (28) | Not reported | Not reported (placebo-controlled) | Placebo-controlled, 3-day course | n = 20 healthy volunteers | No commercial affiliation identified (academic) | Moderate concern from unreported allocation and blinding, of indeterminate direction; no commercial interest identified. |
| Bedada 2017, chlorzoxazone (29) | Not applicable (sequential) | Open-label | Two-period sequential: control period followed by 10-day piperine period | n not reported in the accessible record | No commercial affiliation identified (academic) | Moderate to substantial concern. An open-label sequential design with a fixed period order cannot exclude period effects, and the direction of any such effect is unknown. |
| Bedada 2017, diclofenac (4) | Not applicable (sequential) | Open-label | Two-period sequential, as above | n = 12 healthy volunteers | No commercial affiliation identified (academic) | Moderate to substantial concern of indeterminate direction, as above. |
| Bedada 2017, carbamazepine (7) | Not applicable (sequential) | Open-label | Two-period sequential, as above | n = 12 healthy volunteers | No commercial affiliation identified (academic) | Moderate to substantial concern of indeterminate direction, as above. |
| Bedada 2017, fexofenadine (30) | Not applicable (sequential) | Open-label | Two-period sequential, as above | n = 12 healthy volunteers | No commercial affiliation identified (academic) | Moderate to substantial concern of indeterminate direction, as above. |
| Hussaarts 2019 (32) | Randomized | Not reported | Three-period crossover (tamoxifen alone, with curcumin, with curcumin plus piperine) | n = 15 patients with breast cancer | No commercial affiliation identified (academic hospital; no conflicts declared) | Low concern. The only curcumin–piperine combination study with a curcumin-alone arm, and therefore the only one that isolates piperine’s contribution within a combination; conducted in patients, and its result (a decrease) runs counter to the expectation of enhancement. |

**Summary of the appraisal**

Three observations follow from the table and are reflected in Section 5 of the manuscript.

1. On the nutrient side, each of the three piperine-manufacturer-affiliated studies reporting enhanced exposure carries a sponsorship concern acting in the direction of enhancement, and none was randomized in the accessible record. The studies reporting no enhancement are better designed on the items appraised here (two randomized and blinded), but their samples are modest (6–30 participants in the pharmacokinetic analyses), and one carries a sponsorship concern acting in the opposite direction. The 2025 three-arm study (34) sits outside both groups: its authors’ employer markets the piperine-free comparator, so its concern also runs toward understating piperine, while parallel arms of four and five participants, an unexplained near-absence of absorption in the low-piperine arm, and piperine amounts far above supplement doses limit what it can settle. The asymmetry in sponsorship is real, but it is not the only difference between these groups: era, analytical method and piperine dose also differ, as the manuscript states.

2. On the drug side, the concerns are of design rather than of sponsorship for most studies. Ten of the fourteen have no identified commercial interest, and the four studies from a single group (29, 4, 7, 30) share an open-label sequential design and should not be counted as four independent replications. Even so, increased exposure is reported by five research groups with no identified commercial interest, including in a randomized placebo-controlled crossover study (27) and in patients at steady state (9, 8). The conclusion that piperine increases exposure to co-administered drugs, most of them substrates of CYP3A4, CYP2C9, CYP2E1, or P-glycoprotein, does not depend on the patent-holding group’s studies (24–26).

3. The two studies that do not report increased exposure (31, 32) are among the better designed, and neither can be explained by sponsorship: one has manufacturer-affiliated co-authors and nonetheless reported a null result, and the other has no identified commercial interest and declared no conflicts. This is the basis for the manuscript’s statement that the direction of an interaction cannot be assumed.

Limitations of this appraisal. It was performed by the sole author, without duplicate assessment. It relies on the records accessible to the author, so "not reported" may reflect the abstract or the available full text rather than the conduct of the study; four entries (1, 18, 24, 29) lack a participant count for this reason. Supply of study material by a manufacturer was not assessed for any study. Selective non-reporting could not be appraised, as no protocol or registration record was located for most of the older studies; where a judgment refers to selective emphasis, it concerns the weight placed on one reported arm, not unreported outcomes.
